# Yishen Xiaoke Formula Delays Renal Fibrosis in Rats with Diabetic Nephropathy by Suppressing the Transforming Growth Factor-Beta 1/Notch Signalling Pathway

**DOI:** 10.5812/ijpr-171242

**Published:** 2026-09-23

**Authors:** Shanshan Chang, Xuan Zheng, Qiang Chen, Xuan Liu, Jianhong Zhang

**Affiliations:** 1 Department of Pharmacy, Tianjin Fourth Central Hospital Tianjin China; 2 Department of Traditional Chinese Medicine, Tianjin Fourth Central Hospital Tianjin China

**Keywords:** Diabetic Nephropathy, Renal Fibrosis, Transforming Growth Factor-beta 1, Signal Transduction

## Abstract

**Background:**

Yishen Xiaoke Formula (YSXKF), derived from the classical prescription Yuquan San, has been used clinically to treat diabetic nephropathy (DN) in patients with qi-yin deficiency syndrome.

**Objectives:**

This study investigated whether YSXKF delays renal fibrosis progression in DN rats by regulating the transforming growth factor-beta 1 (TGF-β1)/Notch signalling pathway.

**Methods:**

Seventy-five male Sprague Dawley rats were included and randomly assigned to a control cohort (15 rats) or a model cohort (60 rats). DN was induced by unilateral nephrectomy, a high-fat/high-sucrose diet, and streptozotocin injection. DN rats were treated with YSXKF, irbesartan, or YSXKF combined with TGF-β1 activation for 8 weeks. After 8 weeks of intervention, 24-h urinary total protein (24-h UTP), circulating TGF-β1 levels, fasting blood glucose (FBG), and serum creatinine (Scr) were evaluated. Masson staining was performed to assess renal pathological changes, and quantitative reverse transcription polymerase chain reaction and immunohistochemistry were conducted to assess the levels of TGF-β1, Jagged1, and Hes1 in renal tissue.

**Results:**

Compared with DN rats, YSXKF treatment significantly reduced 24-h UTP excretion, circulating TGF-β1 levels, FBG, and Scr. Histological analysis revealed attenuated glomerulosclerosis and interstitial fibrosis. YSXKF decreased renal mRNA and protein expression of TGF-β1, Jagged1, and Hes1. Activation of TGF-β1 markedly reversed these protective effects and restored Notch pathway activation.

**Conclusions:**

YSXKF delays renal fibrosis progression in DN rats by downregulating TGF-β1 expression and inhibiting Jagged1/Hes1 activation in the Notch signaling pathway, thereby reducing extracellular matrix deposition.

## 1. Background

Diabetic nephropathy (DN) is a severe microvascular complication of diabetes mellitus and has emerged as the predominant cause of end-stage renal disease (1, 2). Globally, approximately 20%–40% of individuals with diabetes ultimately progress to DN (3). Renal fibrosis is recognised as the central pathological hallmark of DN and is characterised by excessive accumulation of extracellular matrix (ECM) in the glomerular basement membrane and tubulointerstitium, ultimately resulting in a progressive decline in renal function (4). Current evidence suggests that transforming growth factor-beta (TGF-β) is a pivotal cytokine driving DN-related fibrosis (5). Transforming growth factor-beta 1 (TGF-β1) activates downstream Smad signalling by binding to its receptors and upregulating the expression of ECM components such as fibronectin and collagen (6). Recent investigations have demonstrated that TGF-β signalling does not act alone but exhibits crosstalk with multiple signalling pathways; among these, the Notch signalling cascade plays a pivotal role in DN development (7, 8). In a high-glucose environment, TGF-β1 can increase the expression of the Notch ligand Jagged1, which activates receptors and promotes transcription of the downstream target gene Hes1, forming a TGF-β/Notch positive-feedback regulatory loop that jointly promotes the progression of renal fibrosis (9).

From the perspective of traditional Chinese medicine (TCM), DN belongs to the category of ‘consumptive thirst kidney disease’, with its basic pathogenesis being prolonged consumptive thirst leading to deficiency of both qi and yin as the root cause, and blood stasis and turbid phlegm obstructing collaterals as the manifestation (10). Recent research suggests that the syndrome of ‘qi and yin deficiency’ is associated with a chronic inflammation–fibrosis-susceptible state, in which qi deficiency leads to decreased organ function and metabolic disorders, whereas yin deficiency aggravates inflammatory reactions and oxidative stress damage (11). Therefore, therapies aimed at replenishing vital energy, restoring yin balance, improving blood circulation, and promoting meridian patency have become important therapeutic principles in the TCM management of DN.

Yishen Xiaoke Formula (YSXKF) originates from the classical prescription ‘Yuquan San’ recorded in Ye Tianshi’s Zhongfutang Selected Good Prescriptions and has been used clinically for the treatment of DN, particularly in patients with the TCM syndrome of qi and yin deficiency (12). In this formula, *Astragalus membranaceus* and *Dioscorea opposita* benefit qi, strengthen the spleen, and consolidate the kidney; *Rehmannia glutinosa*, *Pueraria lobata*, and *Ophiopogon japonicus* nourish yin and generate fluids; *Schisandra chinensis* and *Eleutherococcus senticosus* supplement the kidney and astringe essence; *Carthamus tinctorius* is traditionally used to improve blood circulation and alleviate blood stasis, whereas corn silk facilitates diuresis and helps relieve oedema. Recent pharmacological studies indicate that *Astragalus* polysaccharides can mitigate renal fibrosis by modulating the TGF-β/Smad pathway (13). Experimental studies have demonstrated that Hydroxysafflor yellow A exerts renoprotective effects in DN by attenuating oxidative stress, suppressing inflammatory responses, and reducing renal fibrosis–related injury (14). However, the specific molecular mechanism by which YSXKF delays renal fibrosis in DN, particularly its regulatory effect on the TGF-β/Notch signalling cascade, remains to be elucidated.

## 2. Objectives

Based on these findings, this study used the internationally recognised DN rat model induced by left nephrectomy combined with streptozotocin (STZ) (15) to investigate the modulatory role of YSXKF in the TGF-β1/Notch signalling pathway and the underlying molecular mechanisms by which it delays renal fibrosis, thereby providing experimental evidence supporting the clinical utility of this formula.

## 3. Methods

### 3.1. Experimental Animals

Seventy-five specific pathogen-free (SPF) male Sprague Dawley rats (aged 8 weeks and weighing 260 ± 20 g) were supplied by Beijing Huafukang Biotechnology Co., Ltd., which holds Animal Production License No. SCXK [Jing] 2019 - 0008. The rats were acclimated and housed in a dedicated SPF facility under strictly regulated environmental conditions, including an ambient temperature maintained at 20°C–26°C. Standard chow and water were provided ad libitum throughout the experimental period. All animal experimental procedures were performed in strict accordance with the laboratory animal care and use guidelines of Tianjin.

### 3.2. Drugs and Reagents

The YSXKF composition was as follows: *Astragalus membranaceus* (20 g), *Carthamus tinctorius* (10 g), *Pueraria lobata* (15 g), *Rehmannia glutinosa* (15 g), *Ophiopogon japonicus* (10 g), *Schisandra chinensis* (6 g), *Eleutherococcus senticosus* (9 g), *Dioscorea opposita* (15 g), corn silk (20 g) and *Glycyrrhisa uralensis* (6 g). All Chinese herbal pieces were purchased from Hebei Qiyitang Pharmaceutical Co., Ltd. and authenticated by the Department of Chinese Medicine Identification, Tianjin University of TCM. The standard decoction process involved soaking the compound in water at a proportion of 1:8 (w/v) for 0.5 h, boiling for 30 min, filtering, adding water at a proportion of 1:6 (w/v) to the residue and decocting for another 30 min, combining the two filtrates, concentrating to 1.0 g crude drug/mL and storing at 4°C. High-performance liquid chromatography (HPLC) fingerprinting was used to ensure inter-batch quality consistency. Representative chromatographic fingerprints were established for each batch, and major characteristic peaks corresponding to the principal herbal components were consistently detected. Inter-batch similarity was evaluated using the TCM Chromatographic Fingerprint Similarity Evaluation System, with a similarity coefficient > 0.90 considered acceptable. The chromatographic profiles demonstrated good consistency among different batches of YSXKF used in this study.

Irbesartan tablets (150 mg/tablet, batch number: EA3699) were purchased from Sanofi Pharmaceutical Co., Ltd. STZ (batch number: 102790937) and recombinant human TGF-β1 (Cat. No. T7039) were purchased from Sigma. A high-energy diet rich in fat and sugar (60% basic feed + 20% sucrose + 10% lard + 10% egg yolk powder) was customised by Beijing Huafukang Biotechnology Co., Ltd. The main instruments included an automatic biochemical analyser (Hitachi 7600, Japan), a quantitative reverse transcription polymerase chain reaction (RT-qPCR) instrument (Applied Biosystems 7500, USA), an optical microscope (Olympus BX53, Japan), a paraffin microtome (Leica RM2235, Germany) and a high-speed refrigerated centrifuge (Eppendorf 5810R, Germany).

### 3.3. Diabetic Nephropathy Model Establishment and Grouping

The DN model was established using the STZ combined with unilateral nephrectomy method (15, 16). After 1 week of adaptive feeding, 15 rats were designated as the control group, and the remaining 60 rats underwent left nephrectomy as follows: intraperitoneal anaesthesia with 2% sodium pentobarbital (40 mg/kg), left lumbar skin preparation and disinfection, a 1.5-cm incision, left kidney separation, renal pedicle ligation and resection, and incision closure. Penicillin (40,000 U/rat) was injected intraperitoneally for 3 consecutive days postoperatively to prevent infection.

One week postoperatively, rats were switched to a high-fat, high-sugar diet. After 4 weeks, following a 12-h fast, STZ was injected intraperitoneally at 30 mg/kg (dissolved in 0.1 mol/L citrate buffer at pH 4.2). After 72 h, tail-vein blood glucose was measured. Successful establishment of the DN model was predefined as fulfilment of all the following criteria simultaneously: 1) fasting blood glucose ≥ 16.7 mmol/L at 72 h after STZ injection; 2) a ≥ 50% increase in 24-h urine volume compared with pre-modelling baseline levels; and 3) a ≥ 2-fold increase in 24-h urinary protein excretion compared with pre-modelling baseline levels. Animals that failed to meet any of these criteria were excluded before treatment allocation.

A total of 60 successfully modelled rats were randomly allocated into four experimental groups (n = 15 each): the model group, YSXKF group, irbesartan group (positive control) and YSXKF + TGF-β1/Notch pathway activator group.

Based on the human-to-rat dose conversion coefficient of 0.018, rats in the YSXKF group were administered YSXKF by oral gavage at a daily dose of 11.34 g/kg, and rats in the irbesartan group received irbesartan at a dosage of 13.5 mg/kg/day by oral gavage. Rats in the YSXKF + TGF-β1/Notch pathway activator group received YSXKF (11.34 g/kg/day, gavage) in combination with recombinant TGF-β1, administered by tail-vein injection at 2 μg/kg once weekly for 8 weeks to activate the TGF-β1/Notch signalling pathway. Purified water was provided to the control and model groups in volumes matching those administered to the treatment groups. All interventions were administered continuously for 8 weeks.

During the intervention period, 3 rats from the model group, 1 rat from the YSXKF group, and 2 rats from the irbesartan group died. No deaths occurred in the YSXKF + TGF-β1/Notch activator group. Consequently, the final numbers of animals included in the analyses were 12, 14, 13 and 15 in the model, YSXKF, irbesartan and YSXKF + TGF-β1/Notch activator groups, respectively. No additional animals were excluded after group allocation for experimental, biochemical, histological, molecular or statistical reasons.

#### 3.3.1. Randomisation and Bias Control

Randomisation at all stages (pre-modelling, post-modelling grouping and pre-intervention assignment) was performed using a randomly generated allocation sequence created in Microsoft Excel by a researcher not involved in subsequent experimental procedures to ensure allocation concealment. To minimise potential bias, cages were systematically rotated on the rack weekly to avoid environmental gradients in light, ventilation and temperature. The daily order of gavage administration, sample collection and routine observations was randomised. A single-blinded design was implemented for outcome assessment. After sample collection, all blood, urine and tissue specimens were assigned anonymised identification codes by an investigator not involved in laboratory analyses. Personnel performing biochemical assays [24-h urinary total protein (24-h UTP), FBG, serum creatinine (Scr), and serum TGF-β1], RT-qPCR experiments, immunohistochemical staining and quantification, histopathological evaluation, image acquisition, image analysis and statistical analyses were blinded to group allocation until completion of data analysis. For histological and immunohistochemical assessments, slides were independently evaluated by two investigators blinded to the treatment groups. Representative images included in figures were selected from coded datasets before unblinding. Key measurements included internal technical replicates, and consistency between independent evaluators exceeded 95%. Group allocation codes were not revealed until all primary analyses had been completed.

#### 3.3.2. Sample Size Calculation

The number of animals allocated to each experimental group was determined a priori based on power analysis. Based on a pilot study and previous literature investigating similar endpoints in the STZ-established DN rat model (17), we anticipated a large effect magnitude (Cohen’s d = 0.9 - 1.2) for the primary therapeutic intervention compared with the model group. The calculation was performed for independent samples using a two-tailed Student’s t-test (G*Power 3.1 software). Parameters were set for a confirmatory study aiming to control alpha (α) at 0.05 and β at 0.20 (statistical power of 80%). With an assumed effect size (d) of 1.0, the analysis indicated a minimum requirement of 10 rats per group. To account for potential attrition due to model establishment (e.g. surgical complications, severe hyperglycaemia) and to ensure robust statistical analysis across multiple secondary endpoints, the sample size was increased. Consequently, 60 rats were initially selected for modelling, with a targeted final allocation of 12 - 15 rats per intervention group post-modelling, as reflected in the final group sizes (model: n = 12, YSXKF: n = 14, Irbesartan: n = 13, YSXKF + TGF-β1/Notch activator: n = 15). The experiment was conducted once as a complete, independent cohort; however, key measurements (e.g. collagen volume fraction [CVF], qPCR) included internal technical replicates (e.g. 10 random fields per section, duplicate PCR reactions) to ensure reliability.

### 3.4. Specimen Acquisition and Testing

#### 3.4.1. Biochemical Index Detection

After the final administration, 24-h urine samples were collected in metabolic cages from individually housed rats, centrifuged (5 min; 1,500 rpm), and UTP was assessed using the Coomassie Brilliant Blue assay. After 12 h of fasting, rats were anaesthetised with 2% sodium pentobarbital. After abdominal aortic blood collection, samples were centrifuged (10 min; 3,000 rpm) to isolate serum. FBG and Scr were measured using an automated biochemical analyser, whereas serum TGF-β1 levels were determined by enzyme-linked immunosorbent assay (Fankewei, batch No. 202403).

#### 3.4.2. Renal Pathological Examination

The right kidney was harvested and fixed in 4% paraformaldehyde for 24 h and then subjected to routine tissue processing, clearing, paraffin embedding, and sectioning into 3-μm slices. Masson’s trichrome staining was performed to assess renal fibrotic changes, in which collagen deposition was stained blue and muscle tissue was stained red. For quantitative analysis, 10 randomly selected microscopic fields (200× magnification) from each section were analysed, and CVF was assessed using Image-Pro software (Plus 6.0).

#### 3.4.3. Quantitative Reverse Transcription Polymerase Chain Reaction

Renal RNA was extracted using TRIzol^™^ reagent and reverse-transcribed into complementary DNA. Primer information is provided in Table 1; RT-qPCR was performed, and gene expression levels were normalised to β-actin and analysed according to the 2^-ΔΔCt^ method.

**Table 1.** Primer Sequences

Genes and Primer Sequence (5'→3')	Product Length (bp)
**β-actin**	200
F: CGCGAGTACAACCTTCTTGC	
R: ATACCCACCATCACACCCTG	
**TGF-β1**	123
F: GCAACAATTCCTGGCGTTACC	
R: CCTGTATTCCGTCTCCTTGGTT	
**Jagged1**	88
F: CCTCCAACGATACGCCTGAAG	
R: TTGCCTCCTGACTCGCTCTT	
**Hes1**	80
F: GGCCAATTTGCTTTCCTCATCC	
R: CCGCTGTTGCTGGTGTAGAC	

#### 3.4.4. Immunohistochemical Detection

Paraffin-embedded kidney sections were routinely deparaffinised and rehydrated, after which antigen retrieval was performed by heating in citrate buffer. Endogenous peroxidase activity was inactivated by incubation with 3% hydrogen peroxide. The tissue sections were then incubated with antibodies targeting TGF-β1 (1:200), Jagged1 (1:200) and Hes1 (1:100). After thorough washing, the samples were incubated with secondary antibodies for 0.5 h. Signal development was achieved using 3,3′-diaminobenzidine, followed by haematoxylin nuclear counterstaining.

### 3.5. Outcome Measures and Hierarchy of End Points

To improve methodological transparency and comply with ARRIVE 2.0 guidelines, outcomes in this study were predefined and categorised before data analysis. The primary outcome was 24-h UTP, as it represents the most direct and sensitive marker of renal damage and disease progression in DN. Secondary functional outcomes included serum TGF-β1, FBG and Scr, reflecting systemic metabolic status, fibrotic activity and renal functional impairment, respectively. Secondary structural outcomes included renal pathological changes assessed by Masson staining and CVF. Exploratory mechanistic outcomes included renal abundances of TGF-β1, Jagged1 and Hes1 measured by RT-qPCR and immunohistochemistry, aimed at investigating involvement of the TGF-β1/Notch signalling pathway. All outcome assessments were performed according to the same predefined protocol across groups.

### 3.6. Statistical Analysis

Statistical analysis was performed using SPSS 21.0 software. All quantitative data were presented as mean ± standard deviation. Before statistical comparisons, the Shapiro–Wilk test was used to assess normality, and Levene’s test was used to assess homogeneity of variance among groups. Data meeting the requirements for parametric testing were analysed by one-way analysis of variance, and intergroup differences were further examined using Tukey’s honest significant difference procedure. For datasets that did not meet parametric assumptions, statistical significance was assessed using the Kruskal–Wallis H test. The primary outcome of this study was predefined as 24-h UTP, with other biochemical, histological and molecular parameters considered secondary or exploratory outcomes. Two-sided tests were used, and statistical significance was defined as P < 0.05, with adjustment to minimise type I error arising from multiple testing. No formal adjustment for multiplicity across different outcome domains was applied, as this study was designed as an exploratory preclinical investigation; however, secondary and mechanistic outcomes were interpreted with caution.

## 4. Results

### 4.1. General Observations of Rats in Different Groups

During the experimental period, all 15 rats in the control group survived and maintained a good general condition, with normal growth, glossy fur, normal activity, and stable mental status. Three rats died in the model group, one rat died in the YSXKF group, and two rats died in the irbesartan group, whereas no mortality was observed in the YSXKF + TGF-β1/Notch pathway activator group. Compared with the control group, animals in all model-induced groups exhibited typical diabetic manifestations, including polydipsia, polyuria, weight loss, dull and yellowish fur, and reduced activity. As the experiment progressed, these symptoms became more severe in the model group. In contrast, rats treated with YSXKF or irbesartan showed varying degrees of improvement, as evidenced by a better general condition, increased activity, and improved fur appearance. Although the YSXKF + TGF-β1/Notch pathway activator group also showed partial improvement compared with the model group, the extent of improvement was less pronounced than that in the YSXKF group, suggesting that upregulation of the TGF-β1/Notch signalling pathway partially attenuated the protective effects of YSXKF.

### 4.2. Effects of Yishen Xiaoke Formula on Biochemical Indices in Diabetic Nephropathy Rats

As illustrated in Figure 1, the model group showed significantly higher levels of 24-h UTP, serum TGF-β1, FBG, and Scr than the control group. After 8 weeks of administration, all indices in the YSXKF group were significantly improved compared with those in the model group: 24-h UTP decreased by 31.9% (t = 3.255, P = 0.031, 95% CI: 1.66 - 20.93, Figure 1A), serum TGF-β1 decreased by 45.0% (t = 5.773, P = 0.0045, 95% CI: 125.82 - 358.98, Figure 1B), FBG was reduced by 33.3% (t = 3.189, P = 0.0335, 95% CI: 0.37 - 19.36, Figure 1C), and Scr declined by 27.5% (t = 3.176, P = 0.0339, 95% CI: 1.23 - 80.99, Figure 1D). Compared with the irbesartan group, the YSXKF group exhibited greater reductions in 24-h UTP, TGF-β1, and Scr and also showed a hypoglycaemic effect. Relative to the YSXKF group, rats in the YSXKF + TGF-β1 activator group exhibited significantly increased levels of 24-h UTP, serum TGF-β1, FBG, and Scr. Collectively, these results suggest that activation of TGF-β1 markedly attenuated the renoprotective effects of YSXKF and partially reversed its beneficial effects on renal function and metabolic parameters.

**Figure 1.**
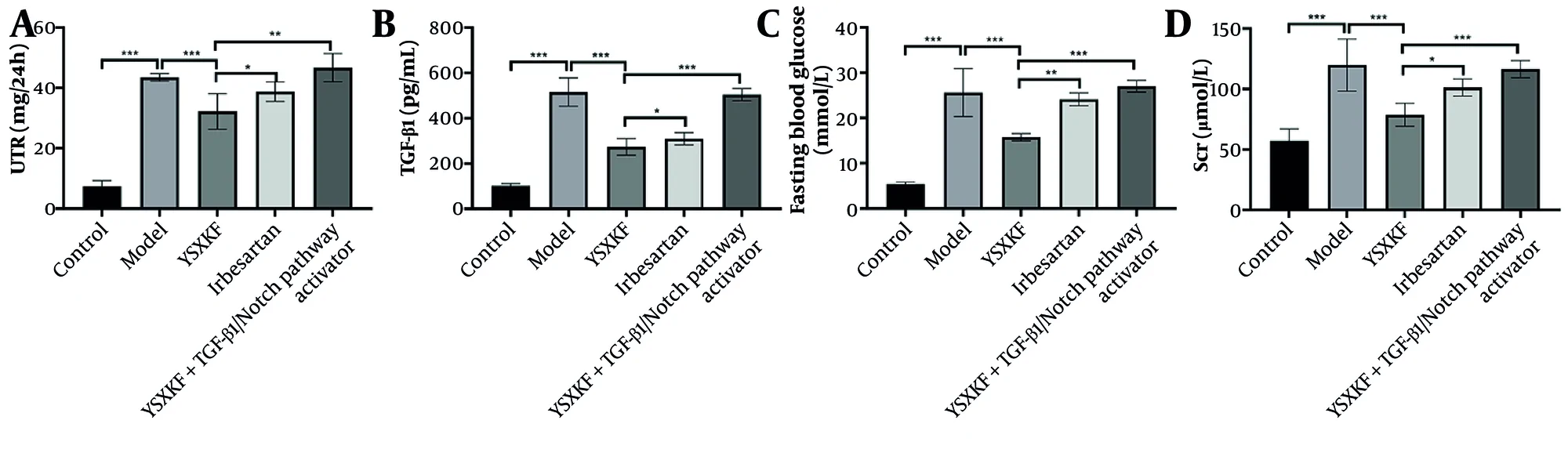
Comparison of biochemical indices in each group. (A) 24-h UTP; (B) serum TGF-β1; (C) fasting blood glucose; (D) Scr. Data are expressed as mean ± SD, n = 12 - 15. *P < 0.05, **P < 0.01, ***P < 0.001.

### 4.3. Effects of Yishen Xiaoke Formula on Renal Pathological Changes in Diabetic Nephropathy Rats

As shown in Figure 2, the control group maintained normal glomerular morphology and exhibited little evidence of interstitial collagen deposition; the model group exhibited marked glomerular hypertrophy, accompanied by expansion of the mesangial matrix and thickening of the basement membrane. Tubular epithelial cells showed obvious vacuolar degeneration, whereas the interstitium displayed extensive deposition of blue-stained collagen fibres (CVF: 32.5% ± 4.2% vs the control group 6.8% ± 1.3%). Pathological damage was significantly reduced in the YSXKF and irbesartan groups, with CVFs of 18.7% ± 3.1% and 22.4% ± 3.6%, respectively. Compared with the YSXKF group, the YSXKF + TGF-β1 activator group exhibited significantly aggravated renal pathological injury, as evidenced by glomerular hypertrophy, mesangial matrix expansion, and increased collagen fibre deposition in the renal interstitium, along with a significantly elevated CVF. These findings indicate that activation of TGF-β1 partially abolished the protective efficacy of YSXKF against renal fibrosis, further supporting the involvement of the TGF-β1/Notch signalling cascade in the renoprotective mechanism of YSXKF.

**Figure 2.**
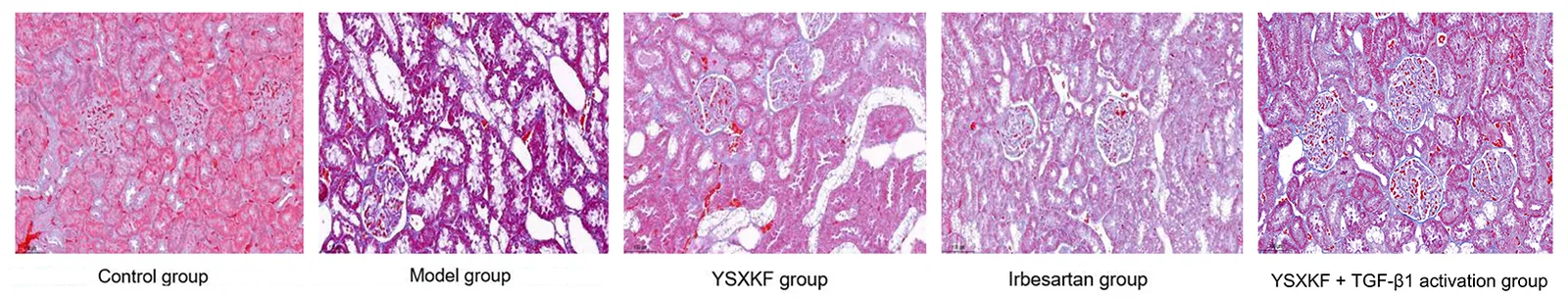
Microscopic observation of rat renal tissue pathological morphology (Masson staining, ×200). Note: Collagen fibers appear blue, and muscle fibers appear red.

### 4.4. Effects of Yishen Xiaoke Formula on TGF-β1, Jagged1, and Hes1 Expression in Renal Tissue

RT-qPCR analysis (Figure 3) demonstrated that renal TGF-β1, Jagged1, and Hes1 mRNA levels in the model group were increased by 1.80-, 2.06-, and 1.98-fold, respectively, compared with those in the control group. Following YSXKF intervention, their expression decreased by 21.7%, 30.8%, and 37.8%, respectively, versus the model group, and the reductions in Jagged1 and Hes1 were greater than those observed in the irbesartan group.

**Figure 3.**
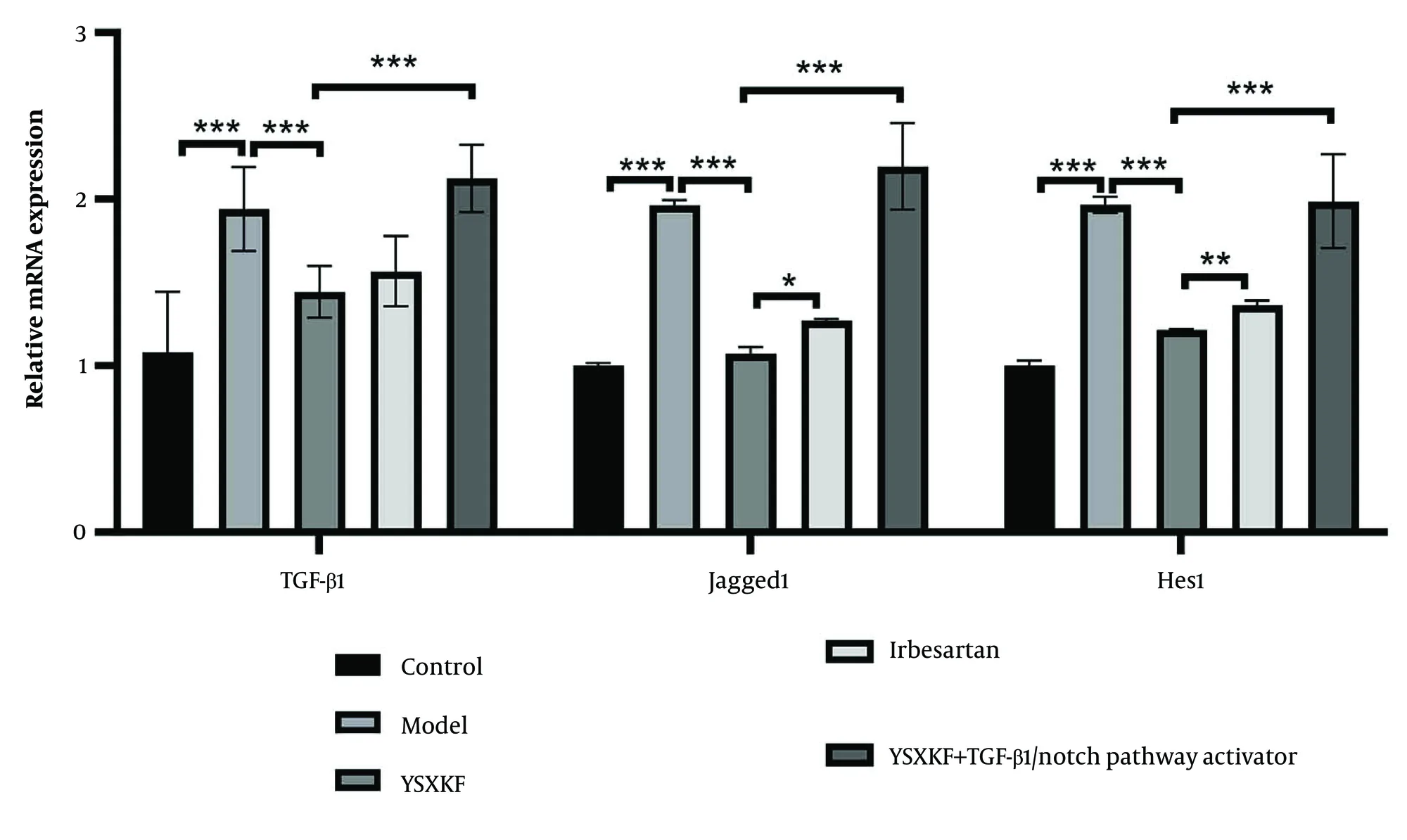
Relative mRNA abundances of TGF-β1, Jagged1, and Hes1 in rat renal tissue. Data are expressed as mean ± SD, n = 12 - 15. *P < 0.05, **P < 0.01, ***P < 0.001.

Immunohistochemical results (Figure 4) were consistent with the mRNA expression trends, with strong positive expression (brown-yellow granules) of TGF-β1, Jagged1, and Hes1 observed in glomerular mesangial areas, tubular epithelial cells, and interstitium in the model group. Compared with the YSXKF group, the YSXKF + TGF-β1 activator group showed significantly increased abundances of TGF-β1, Jagged1, and Hes1 in renal tissues, along with stronger positive staining intensity and a larger positive staining area (Figures 3 and 4). These results suggest that exogenous TGF-β1 activation reactivated the TGF-β1/Notch signalling cascade and partially reversed the suppressive effect of YSXKF on pathway-related molecules.

**Figure 4.**
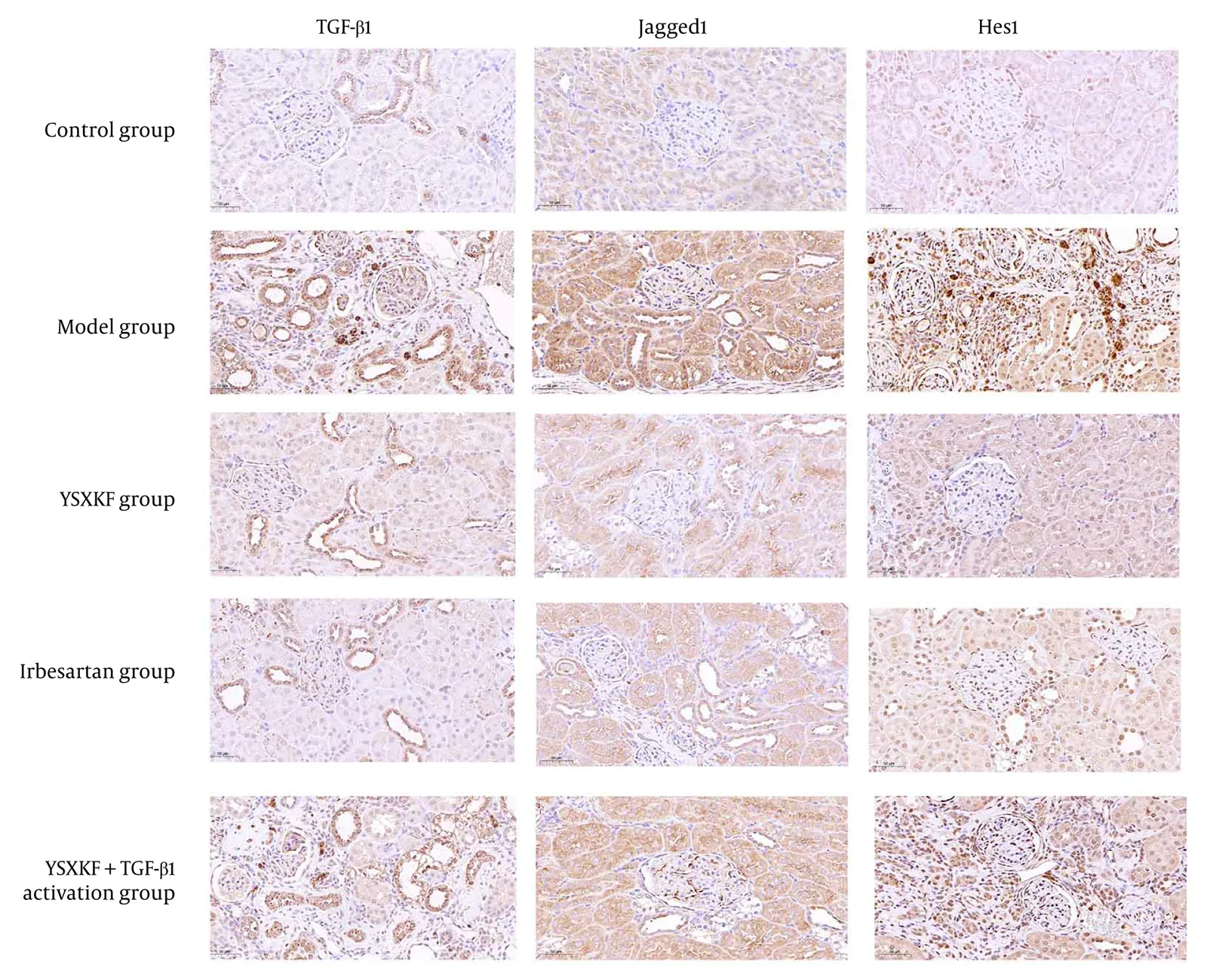
Protein expression of TGF-β1, Jagged1, and Hes1 in rat renal tissue (immunohistochemistry, ×400). Note: Brown-yellow granules indicate positive expression.

## 5. Discussion

DN has a complex pathogenesis involving multiple processes, including haemodynamic changes, metabolic disorders, inflammatory responses, and fibrosis (17). At the pathophysiological level, metabolic memory induced by chronic hyperglycaemia, together with mitochondrial dysfunction, endoplasmic reticulum stress, and epigenetic alterations, collectively contributes to the initiation and progression of DN. Owing to its high metabolic activity and substantial energy demands, the kidney is particularly vulnerable to disturbances in glucose homeostasis. Pathological processes, including alterations in glomerular haemodynamics, activation of the renin–angiotensin–aldosterone system (RAAS), and inflammatory cell recruitment, act synergistically to promote progressive renal injury, ultimately resulting in irreversible structural and functional impairment (18). This study employed the internationally recognised left nephrectomy combined with the STZ-induction method to establish a DN model, which can simulate the natural course of human DN from hyperfiltration to renal function decline (15, 19). Unilateral nephrectomy increases blood perfusion and the filtration load of the remaining kidney, placing the glomeruli under high pressure and high perfusion. This haemodynamic change acts synergistically with glucotoxicity to accelerate the progression of diabetic renal injury. This model can produce marked glomerulosclerosis and interstitial fibrosis within 8 weeks, more closely resembling the pathological features of human DN than STZ induction alone (20). Additionally, this model can reproduce clinical features of patients with DN, such as proteinuria progression and renal function decline, providing an experimental platform for evaluating drug efficacy.

The present findings demonstrate that YSXKF not only attenuated 24-h UTP excretion and ameliorated renal dysfunction in DN rats, but also effectively alleviated renal fibrosis. Specifically, 24-h UTP decreased by 31.9% compared with the model group, and Scr decreased by 27.5%, suggesting that YSXKF effectively alleviated renal injury and improved renal function in the experimental DN model. Pathological observations further confirmed that YSXKF reduced glomerular hypertrophy, mesangial matrix proliferation, and interstitial collagen deposition, with CVF decreased by 42.5% versus the model group. Compared with the angiotensin (AT) receptor antagonist irbesartan, YSXKF produced larger changes in several efficacy indicators, including a hypoglycaemic effect (FBG decreased by 33.3%), reflecting the multitarget, multipathway therapeutic advantages of TCM. These combined effects may be related to the holistic regulatory effects of Chinese herbal compound formulas, which not only target local renal lesions but also improve systemic metabolic status, thereby fundamentally reducing glucotoxic damage to the kidney.

TGF-β1 is a core mediator of DN fibrosis (5, 21). As a multifunctional cytokine, TGF-β1 plays a dual role in DN: at physiological concentrations, it exerts anti-inflammatory effects and maintains glomerular barrier function, whereas excessive activation under pathological conditions promotes fibrosis. Hyperglycaemia triggers a variety of molecular events that promote excessive synthesis and activation of TGF-β1. Notably, advanced glycation end products (AGEs) can rapidly induce TGF-β/Smad pathway activation through the receptor for AGE-mediated ERK/p38 MAPK signalling axis (6); ATII upregulates TGF-β1 expression through AT1 receptors; and oxidative stress can induce the activation of major transcriptional regulators, such as nuclear factor kappa B and activator protein 1, thereby promoting TGF-β1 gene transcription. This study found that serum TGF-β1 levels in the model group were increased 3.5-fold versus those in the control group, renal tissue TGF-β1 mRNA expression was upregulated 1.8-fold, and protein expression was strongly positive, consistent with literature reports (22). Following YSXKF intervention, TGF-β1 expression was significantly downregulated, with serum levels decreased by 45%, suggesting that this formula may exert antifibrotic effects by inhibiting TGF-β1 production and activation. This inhibitory effect may involve multiple mechanisms, including reducing AGE generation, attenuating oxidative stress, and regulating RAAS activity.

Recent studies have shown that the Notch signalling pathway plays an important role in DN (7, 23). Under normal conditions, Notch signalling in mature kidneys is quiescent and only transiently activated during tissue repair. However, under diabetic conditions, persistent hyperglycaemia, haemodynamic changes, inflammatory factors, and other stimuli lead to abnormal and sustained activation of Notch signalling (24). Overactivated Notch signalling promotes renal fibrosis through multiple mechanisms, including inducing podocyte apoptosis and detachment and disrupting the glomerular filtration barrier; promoting tubular epithelial cell dedifferentiation and mesenchymal transition; activating fibroblast proliferation and ECM synthesis; and forming positive feedback loops with other profibrotic signalling pathways. TGF-β1 can promote the expression of the Notch ligand Jagged1. Following ligand–receptor interaction, γ-secretase cleavage releases the Notch intracellular domain (NICD) from the receptor complex, enabling its nuclear translocation and subsequent binding to RBP-Jκ to initiate transcription of target genes, including Hes1 and Hey1 (25).

Hes1 plays a key role in DN fibrosis; it can downregulate E-cadherin expression and upregulate mesenchymal markers (α-smooth muscle actin and Vimentin) (26). Epithelial-to-mesenchymal transition is considered a key cellular source of renal interstitial fibrosis, with approximately 30%–36% of interstitial myofibroblasts originating from phenotypic transformation of tubular epithelial cells. Additionally, Hes1 can promote fibroblast activation and proliferation, enhancing their ability to synthesise ECM; inhibit matrix metalloproteinase expression, thereby reducing ECM degradation; and induce the expression of profibrotic cytokines (CTGF and PAI-1). This study found that Jagged1 and Hes1 expression in the renal tissue of DN model rats was upregulated by 2.06-fold and 1.98-fold, respectively, with immunohistochemistry showing their main localisation in glomerular mesangial areas, tubular epithelial cells, and the interstitium, consistent with fibrotic lesion sites. These findings suggest that Notch signalling activation is closely related to DN fibrosis.

This study demonstrated that YSXKF can simultaneously downregulate Jagged1 and Hes1 expression, with effects superior to those of irbesartan, suggesting that this formula may delay renal fibrosis by suppressing TGF-β1/Notch signalling activation. Specifically, YSXKF reduced Jagged1 mRNA expression by 30.8% and Hes1 by 37.8%, with even more pronounced decreases at the protein level (50.0% and 47.6%, respectively). To further verify the association between the TGF-β1/Notch signalling pathway and the renoprotective effects of YSXKF, a YSXKF + TGF-β1 activation group was included in this study. Notably, exogenous administration of recombinant TGF-β1 significantly attenuated the beneficial effects of YSXKF. Compared with the YSXKF group, rats receiving TGF-β1 activation exhibited increased 24-h UTP excretion, elevated serum TGF-β1 and Scr levels, aggravated renal pathological injury, and enhanced collagen deposition. Furthermore, the expression levels of TGF-β1, Jagged1, and Hes1 were significantly enhanced following TGF-β1 activation. These findings indicate that activation of TGF-β1 partially restored the suppressive effects of YSXKF on the TGF-β1/Notch signalling pathway and markedly weakened its renoprotective and antifibrotic activities. Therefore, suppression of TGF-β1-mediated Notch signalling appears to be a critical mechanism underlying the therapeutic effects of YSXKF against DN-associated renal fibrosis. The partial rather than complete reversal observed following TGF-β1 activation also suggests that additional signalling pathways may contribute to the therapeutic actions of YSXKF. This inhibitory effect may be achieved through multiple mechanisms, including directly inhibiting TGF-β1 production to reduce Notch signalling activation at the source; interfering with Jagged1 binding to Notch receptors; inhibiting γ-secretase activity to prevent NICD release; promoting NICD ubiquitination and degradation; and upregulating endogenous inhibitors of Notch signalling, such as Numb and Deltex. Notably, positive feedback regulation exists between TGF-β1 and Notch: Notch activation can upregulate TGF-β1 promoter activity via Hes1, thereby further enhancing TGF-β1 expression and forming a vicious cycle (27). Yishen Xiaoke Formula may break this vicious cycle by simultaneously inhibiting both pathways, thereby exerting stronger renoprotective effects.

From a modern pharmacological perspective, multiple active components in YSXKF exert antifibrotic effects, acting synergistically through distinct mechanisms. *Astragalus* polysaccharides have been proven to improve DN by regulating TGF-β/Smad3 signalling, with mechanisms that include inhibiting Smad3 phosphorylation, upregulating Smad7 expression, and regulating Smad cofactors (13). *Astragalus* saponins can also activate AMPK signalling, improve glucose and lipid metabolism, and reduce renal lipid deposition. Safflower yellow A can inhibit high glucose-induced fibronectin and collagen IV expression in mesangial cells, with mechanisms involving antioxidation, anti-inflammation, and improved microcirculation (14). Catalpol in *Rehmannia glutinosa* has hypoglycaemic, antioxidant, and antiapoptotic effects; Ophiopogon polysaccharides can regulate immune function and reduce renal inflammatory responses; and corn silk total saponins have diuretic, antihypertensive, and renal function-improving effects. The synergistic action of these components may be the material basis for the comprehensive efficacy of YSXKF, reflecting the ‘monarch, minister, assistant and guide’ compatibility theory of Chinese herbal compound formulas. The biological pathways investigated in this study and their potential interactions are summarised in Figure 5.

**Figure 5.**
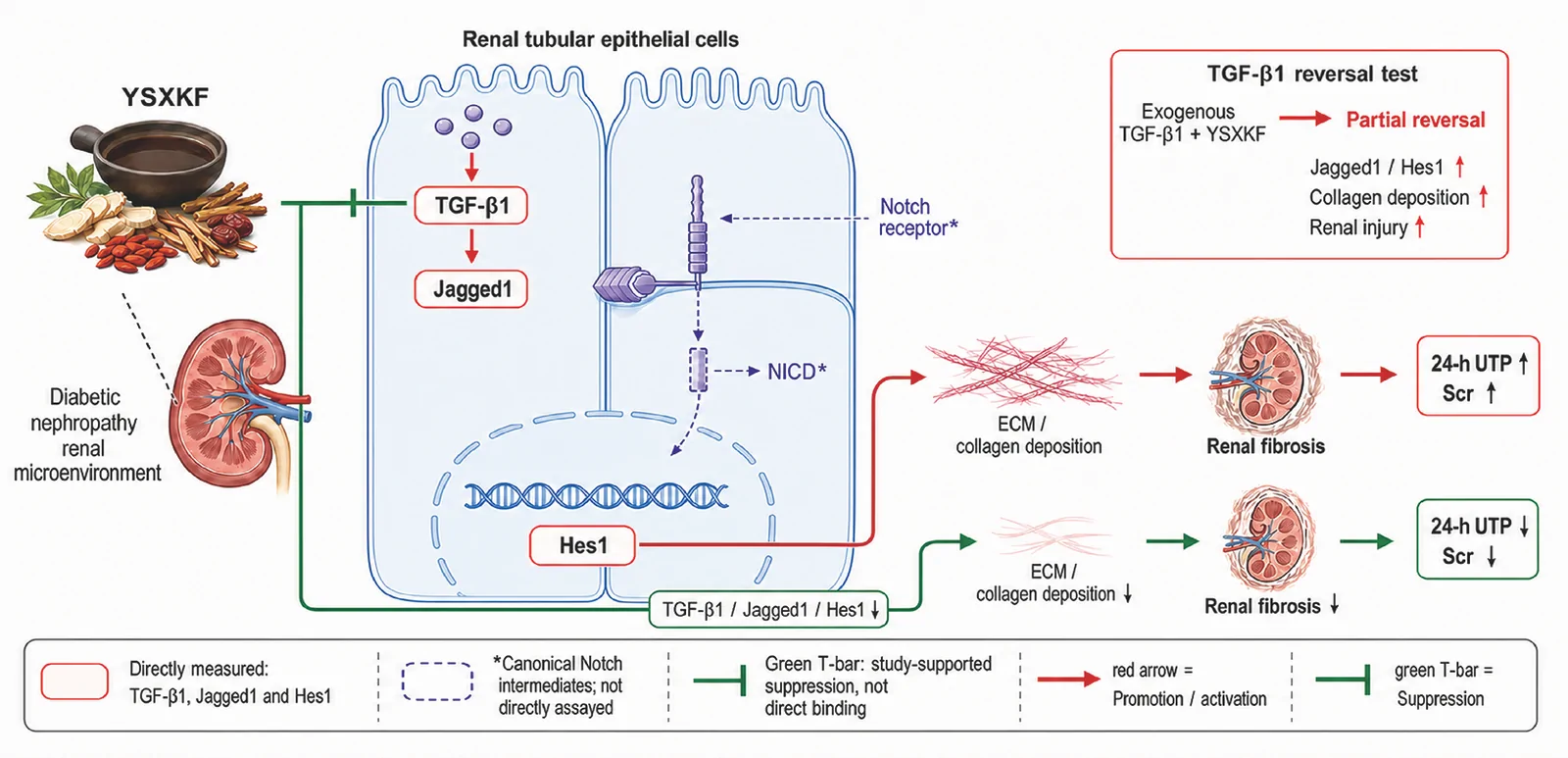
Proposed mechanism of Yishen Xiaoke Formula (YSXKF)-mediated attenuation of renal fibrosis in diabetic nephropathy. YSXKF inhibits TGF-β1 expression and suppresses Jagged1/Hes1 activation in the Notch signalling pathway, thereby reducing extracellular matrix (ECM) deposition, alleviating renal fibrosis, and improving renal function. Activation of TGF-β1 partially reverses the protective effects of YSXKF, resulting in enhanced Jagged1/Hes1 signalling, collagen accumulation, and renal injury. Solid arrows indicate experimentally validated regulatory effects, whereas dashed arrows represent canonical Notch signalling processes not directly examined in this study. Red arrows indicate activation/promotion, and green bars indicate inhibition. YSXKF: Yishen Xiaoke Formula; DN: diabetic nephropathy; TGF-β1: transforming growth factor-beta 1; Jagged1: Jagged Canonical Notch Ligand 1; Hes1: Hairy and Enhancer of Split-1; NICD: Notch Intracellular Domain; ECM: extracellular matrix; 24-h UTP: 24-h urinary total protein; Scr: serum creatinine.

### 5.1. Study Limitations

This study has several limitations. First, only 8-week short-term efficacy was assessed, and long-term effects require verification. Second, dose–response relationship studies were not conducted. Third, exploration of other fibrosis-related pathways, such as Wnt/β-catenin and PI3K/Akt, was lacking. Future studies should extend the observation period, integrate network pharmacology and metabolomic approaches to further elucidate the underlying molecular mechanisms of YSXKF, and conduct high-quality clinical trials to validate its therapeutic efficacy.

### 5.2. Conclusions

YSXKF effectively ameliorated renal impairment and attenuated renal fibrosis in STZ-induced DN rats. Its mechanism of action may involve downregulating TGF-β1 expression, inhibiting Jagged1/Hes1 activation in the Notch signalling pathway, and reducing ECM deposition, thereby delaying DN progression. These findings support the potential clinical use of YSXKF and may facilitate the development of novel therapeutic strategies for DN.

## Data Availability

All data generated or analyzed during this study are included in the article.
